# Characterization of adalimumab Fab variants with the CH1 domain replaced by the Cα1 domain of IgA

**DOI:** 10.1016/j.bbrep.2025.102269

**Published:** 2025-09-19

**Authors:** Rara Sugimoto, Hitomi Nakamura, Masato Kiyoshi, Akiko Ishii-Watabe, Naoko Oda-Ueda, Takatoshi Ohkuri

**Affiliations:** a Faculty of Pharmaceutical Sciences, Sojo University, Japan; b Division of Biological Chemistry and Biologicals, National Institute of Health Sciences, Japan

**Keywords:** Adalimumab, Fab, IgA, Thermal stability, Mutation analysis

## Abstract

Antibody constant domains (C domains) contribute to structural stability. However, studies focusing on Fab fragments with heterologous constant domains are limited. Here, we engineered an IgA-type Fab (Fab_CH1IgA) by replacing the CH1 domain of an IgG1-type adalimumab Fab with the corresponding Cα1 domain of IgA1. Fab_CH1IgA was expressed in CHO cells at approximately 50 mg L^−1^ and purified to homogeneity. DSC showed comparable thermal stability, with *T*_m_ = 74.8 °C for Fab_CH1IgA and 75.2 °C for the IgG1-type Fab. SPR analysis showed similar antigen-binding kinetics, with *K*_D_ = 2.23 n M for Fab_CH1IgA and 1.77 nM for the IgG1-type Fab. Structural analysis identified Pro124 and Tyr211 in the C domain as part of the hydrophobic core-bridging variable and constant domains. Substitution of these residues with their IgG-type counterparts reduced thermal stability, underscoring the critical contribution of V–C domain interactions. Although the sequence identity between the IgG1 and IgA1 constant domains was not particularly high, the CH1 domain in adalimumab Fab could be replaced by the IgA Cα1 domain without markedly compromising stability or activity. These findings highlight cooperative packing at the V–C interface, offering important insights into Fab engineering to enhance function while preserving biophysical integrity, and supporting the design of IgG–IgA chimeric antibodies and novel chimeric Fab formats.

## Highlights

• Fab_CH1IgA, with IgG1 CH1 replaced by IgA1 Cα1, was expressed and retained thermal stability.
• Replacing CH1 with Cα1 preserved the antigen-binding activity of the Fab.
• Cooperative packing at the V–C domain interface is critical for maintaining Fab thermal stability.

## 1 Introduction

Since the 1990s, antibody therapeutics have undergone remarkable development, leading to their widespread adoption as a treatment modality for various diseases [1]. In addition to conventional full-length antibodies, a diverse array of novel formats, including antibody fragments, bispecific antibodies, and antibody–fusion proteins, have been developed to broaden therapeutic options [2]. Among these, antigen-binding fragments (Fab) are particularly noteworthy; because of their smaller molecular size, they exhibit enhanced tissue penetration, and their rapid systemic clearance combined with the absence of an Fc region minimizes nonspecific immune activation [3,4]. Moreover, Fab fragments display superior thermal stability compared with single-chain variable fragments (scFv) [5]. In addition, unlike full-length antibodies that are typically produced in mammalian expression systems, antibody fragments can be efficiently produced in alternative hosts such as yeast or *E. coli*, reducing manufacturing costs [6,7]. Although these features support the growing potential of Fab-based therapeutics [8], most currently approved antibody drugs are in the IgG class [1,9].

On the other hand, the development of therapeutic agents based on novel antibody classes, such as IgA, has attracted considerable attention [10]. Human IgA has two isoforms: IgA1 and IgA2. IgA1 predominantly circulates in the serum as monomeric IgA (mIgA), whereas IgA2 is primarily localized on mucosal surfaces as secretory IgA (sIgA). IgA1, with its elongated hinge region and extensive O-glycosylation, exhibits superior antigen binding and pathogen neutralization, whereas IgA2, particularly the sIgA form, confers enhanced effector function and greater resistance to mucosal bacterial proteases [11,12]. Notably, mIgA binds to FcαRI and attenuates inflammation through FcαRI-mediated inhibition of immunoreceptor tyrosine-based activation signaling in arthritis models [13,14], underscoring the therapeutic potential of IgA in inflammatory disorders. Furthermore, recent studies in the context of COVID-19 demonstrated that sIgA exhibits superior neutralizing activity against SARS-CoV-2 compared with that of IgG, leading to a growing interest in sIgA as a novel modality for respiratory antibody therapy, particularly in the form of aerosolized inhalation agents [15]. Despite these advances, studies focusing on the Fab fragment of IgA remain limited, and its physicochemical properties such as structural stability and antigen-binding activity are unclear.

We previously generated a recombinant Fab derived from adalimumab (Humira®), a fully human monoclonal IgG antibody specific for human TNFα, and conducted studies aimed at improving its physicochemical properties by focusing on the C domain of the Fab, with the general purpose of providing insights applicable to other antibodies. These efforts included the identification of amino acid residues critical for Fab stability [16], enhancement of stability through site-directed mutagenesis [[17], [18], [19]], and the reduction of aggregation propensity by introducing glycosylation [20]. Although numerous studies have examined the C domains of IgG Fab fragments, much less is known about the corresponding domains of other isotypes, such as IgA. Here, we focused on IgA and investigated the physicochemical features of its Cα1 domain. In the present study, to explore novel Fab formats, we engineered a chimeric Fab in which the CH1 domain of the heavy chain in the adalimumab Fab fragment was replaced with the Cα1 domain of IgA1 and evaluated its structural stability and antigen-binding activity.

## 2 Materials and methods

### 2.1 Construction of expression plasmids

The VH gene derived from the heavy-chain V domain of adalimumab, a therapeutic antibody of the human IgG1 subclass, and the Cα1 gene derived from the human IgA1 heavy chain were individually amplified by PCR and subsequently ligated to generate the full-length chimeric heavy-chain gene. The genes encoding the chimeric heavy chain and the κ light chain of adalimumab were each cloned into the pcDNA3.4 vector (Thermo Fisher Scientific, USA) with N-terminal immunoglobulin heavy chain signal sequence (GenBank: QBK47499.1). Mutations were introduced into the heavy chain genes of Fab_CH1IgA via site-directed mutagenesis [17].

### 2.2 Expression and purification of Fab variants

The vectors were transiently transfected into ExpiCHO-S cells using the ExpiFectamine CHO Transfection Kit (Thermo Fisher Scientific, USA) according to the manufacturer's instructions. The cells were cultured for 10 days at 37 °C in an atmosphere of 8 % CO_2_. The cultures were centrifuged at 5000×*g* for 30 min, and the supernatant was collected. Following dialysis against PBS loaded onto a Protein l-Agarose HC column (1.5 × 1 cm; Protenova, Japan) pre-equilibrated with PBS. Elution was carried out with 0.1 M Gly-HCl buffer, pH2.5 and immediately neutralized with 1 M Tris-HCl solution. The Fab-containing fraction was subsequently dialyzed against 50 mM sodium acetate buffer, pH 5.0 (buffer A) and the sample underwent additional purification by cation-exchange chromatography on a resource S column (bed volume, 1 mL; Cytiva, USA) operated on an ÄKTA purifier system (Cytiva, USA). The column was equilibrated with buffer A and proteins were eluted with a linear NaCl gradient (0–0.4 M) in buffer A at a flow rate of 1 mL/min, with a total elution volume of 30 mL.

### *2.3* DSC measurements

DSC measurements were performed using a PEAQ-DSC instrument (Malvern, UK). Thermograms of Fab were monitored from 25 to 100 °C at a scan rate of 90 °C/h. The protein solutions were prepared at concentration of 0.2 mg/mL in 50 mM KH_2_PO_4_ buffer, pH 6.5. The melting temperature (*T*_m_) values were calculated by a standard fitting procedure using an evaluation software using a non-two state model.

### *2.4* Antigen-binding activity measurements

The interaction between adalimumab Fab variants and human TNFα was analyzed using SPR in a Biacore 8K instrument (Cytiva, USA). PBS supplemented with 0.005 % Tween-20 was used as running buffer. The anti-His tag antibody was first immobilized on the CM5 sensor chip (Cytiva, USA). A His-tagged human TNFα reagent (Elabscience, USA) was subsequently injected and captured on the sensor chip. 1 μg/mL (20 nM as a trimer) of TNFα was injected for 90 s, and the aimed capture level was 40 RU. The adalimumab Fab samples were diluted using the running buffer by 2-fold serial dilutions from 64 to 0.5 nM. The contact and dissociation time were 120 s and 300 s, respectively. Regeneration solution was 10 mM Gly-HCl buffer, pH 1.5. Association (*k*_on_) and dissociation (*k*_off_) rate constants were calculated by a global fitting analysis assuming a Langmuir binding model and a stoichiometry of (1:1). The reliability of the kinetic parameters was assessed using the uniqueness value (U-value), with values ≤ 15 regarded as statistically acceptable.

## 3 Results

### 3.1 Preparation of Fab_CH1IgA

The Fab was engineered by replacing the CH1 domain of the IgG1-type adalimumab Fab (wild-type Fab) with the corresponding constant domain (Cα1 domain) from the heavy chain of the IgA1 isotype, generating Fab_CH1IgA. Fig. 1A shows the amino acid sequences of the C domains of the heavy chains from the Fab regions of IgG1 and IgA1, along with a schematic representation of Fab_CH1IgA. The sequence identity between the two domains was not particularly high. Notably, the IgA Cα1 domain contains one additional intramolecular disulfide bond compared with its IgG1 counterpart, representing a distinct structural feature. Fab_CH1IgA was expressed in CHO cells and subsequently purified. Final purification was performed using cation-exchange chromatography, and the elution profile is shown in Fig. 1B. The major elution peak was collected and the fractions were analyzed by SDS–PAGE under both non-reducing and reducing conditions (Fig. 1B). Under non-reducing conditions, a band was observed at approximately 45 kDa (theoretical molecular weight: 47 kDa), corresponding to an intact Fab molecule containing one interchain disulfide bond between the heavy and light chains. Under reducing conditions, bands were observed near the 25 kDa marker, corresponding to the expected molecular weights of the Fab heavy chain (24 kDa) and light chain (23 kDa), respectively. Notably, the bands appeared slightly above the 25 kDa marker, which is consistent with the known characteristic of adalimumab Fab [18]. In addition, size-exclusion chromatography (SEC) analysis of the purified Fab_CH1IgA revealed a single major peak corresponding to the monomeric Fab, indicating that the preparation was homogeneous. These results confirmed that the purified protein was Fab_CH1IgA (Supplementary Fig. S1).

**Fig. 1:**
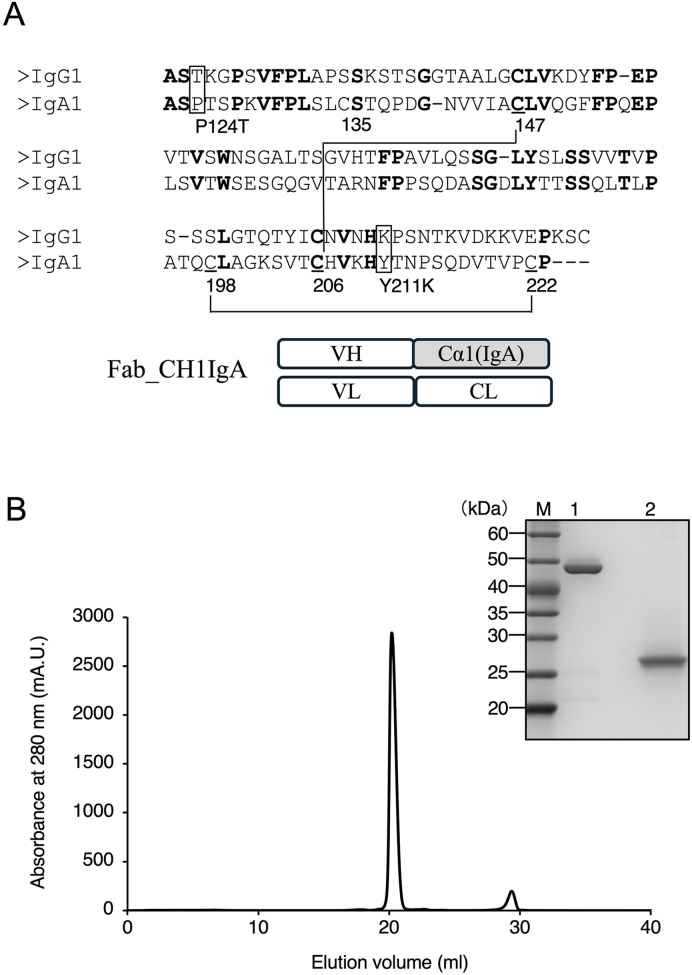
Preparation of Fab_CH1IgA. (A) Amino acid sequences of the CH1 and Cα1 domains of human IgG1 and human IgA1, with identical residues shown in bold, and the schematic representation of Fab_CH1IgA. The point mutation sites and cysteine residue numbers are indicated, and the intradomain disulfide bond is represented by a line. The VH, VL, and CL domains were derived from adalimumab IgG, whereas the CH1 domain was replaced with the Cα1 domain from IgA. (B) Cation-exchange chromatography of Fab_CH1IgA and SDS–PAGE analysis of the eluted fraction from resource S column. Fab_CH1IgA was purified by a resource S column in ÄKTA purifier system. The column was equilibrated with 50 mM sodium acetate buffer, pH 5.0 and eluted with a linear gradient of NaCl from 0 to 0.4 M in equilibration buffer at a flow rate of 1 mL/min and total elution volume of 30 mL. The samples of the eluted fraction were analyzed by 12 % SDS–PAGE. Lane M: protein markers. Lane 1: non-reducing condition, Lane 2: reducing condition.

### 3.2 Secondary structure and thermal stability of Fab_CH1IgA

The circular dichroism (CD) spectrum of Fab_CH1IgA was recorded in 50 mM KH_2_PO_4_ buffer at pH 6.5 (Supplementary Fig. S2). The spectrum was nearly identical to that of the wild-type Fab, indicating that the secondary structure was preserved. The structural stability was further evaluated using DSC. The thermal denaturation midpoint temperature (*T*_m_) of Fab_CH1IgA was determined to be 74.8 °C, which is comparable to that of the wild-type Fab (75.2 °C), as shown in Fig. 2. Although the thermograms appeared approximately Gaussian and symmetrical, the raw DSC data showed slight deviations from an ideal two-state transition. Therefore, a non-two-state model was applied for curve fitting, as it more appropriately accounts for such deviations. These results suggest that replacing the CH1 domain with an IgA-type sequence does not substantially affect the secondary structure and the thermal stability of the adalimumab Fab fragment.

**Fig. 2:**
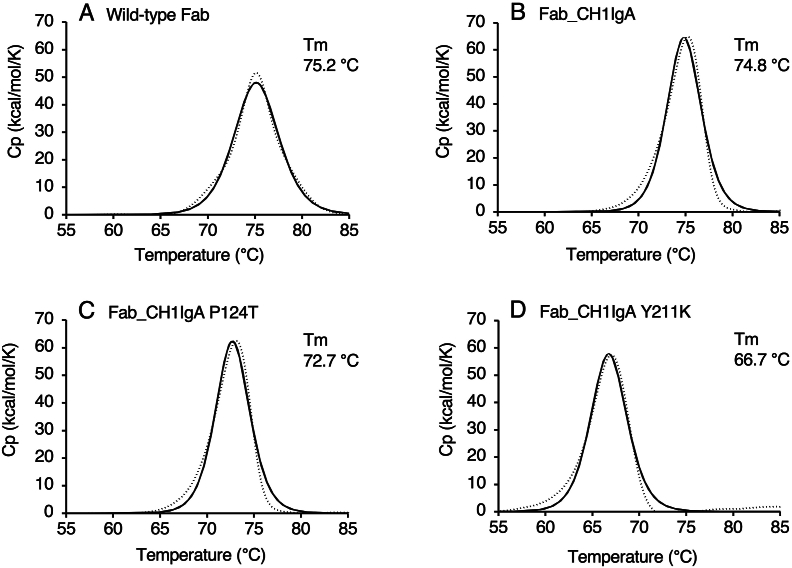
DSC analysis of Fab_CH1IgA variants. Thermograms of the wild-type Fab (A), Fab_CH1IgA (B), Fab_CH1IgA P124T (C), Fab_CH1IgA Y211K (D) were measured between 25 and 100 °C, at a scan rate of 90 °C/h. The Fab variants were adjusted at 0.2 mg/mL in 50 mM KH_2_PO_4_ buffer, pH 6.5. Dashed lines represent the raw data, and solid lines represent the fitted data.

### 3.3 Binding activities of Fab_CH1IgA

**Fig. 3:**
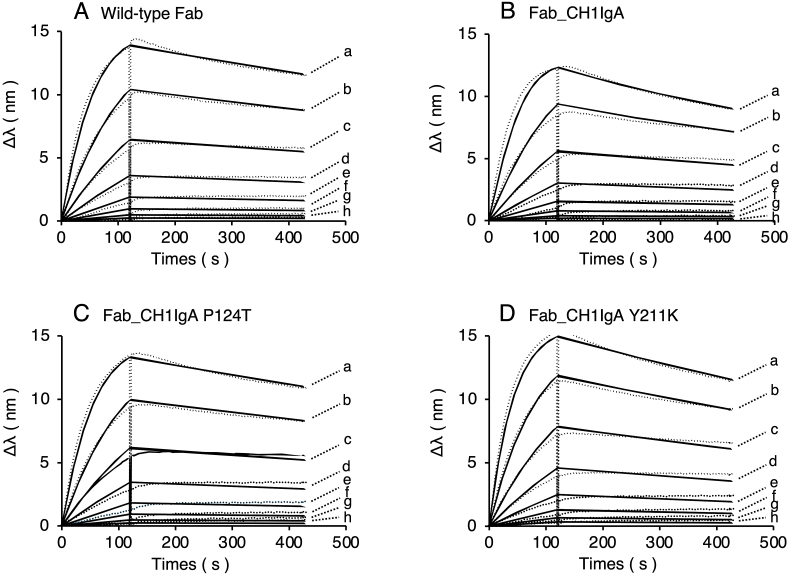
SPR analysis of Fab_CH1IgA variants. The interaction between adalimumab Fab variants, the wild-type Fab (A), Fab_CH1IgA (B), Fab_CH1IgA P124T (C), Fab_CH1IgA Y211K (D) and human TNFα was analyzed by SPR using a Biacore. Sensorgrams are shown for Fab concentrations of 64 nM (a), 32 nM (b), 16 nM (c), 8 nM (d), 4 nM (e), 2 nM (f), 1 nM (g), and 0.5 nM (h). An anti-His tag antibody was immobilized on a CM5 sensor chip, followed by capture of His-tagged human TNFα. Fab samples were injected with contact and dissociation times of 120 s and 300 s, respectively. The chip was regenerated with 10 mM Gly-HCl (pH 1.5), and kinetic parameters (*k*_on_ and *k*_off_) were obtained by global fitting to a 1:1 Langmuir model. Dashed lines represent the raw data, and solid lines represent the fitted data.

The binding activity of the wild-type Fab and Fab_CH1IgA to the antigen TNFα was evaluated by SPR using a Biacore system (Fig. 3). Kinetic analysis was conducted at five Fab concentrations (64–0.5 nM), yielding association (*k*_on_), dissociation (*k*_off_), and equilibrium (*K*_D_) constants (Table 1). The *K*_D_ values for the wild-type and Fab_CH1IgA were 1.77 × 10^−9^ M and 2.23 × 10^−9^ M, respectively. The *k*_on_ and *k*_off_ values remained largely unchanged between the two Fab constructs, indicating that replacing the wild-type CH1 domain with the human IgA1 Cα1 domain did not affect antigen-binding activity.

**Table 1:** Kinetic parameters of the TNFα binding.

	*k*_on_ [1/Ms]	*k*_off_ [1/s]	*K*_D_ [M]	SE (*k*_on_) [1/Ms]	SE (*k*_off_) [1/s]	U-value
Wild-type Fab	3.53 × 10^5^	6.24 × 10^−4^	1.77 × 10^−9^	4.84 × 10^2^	1.73 × 10^−6^	3
Fab_CH1IgA	5.70 × 10^5^	1.27 × 10^−3^	2.23 × 10^−9^	8.52 × 10^2^	2.25 × 10^−6^	3
Fab_CH1IgA P124T	3.53 × 10^5^	6.65 × 10^−4^	1.89 × 10^−9^	8.33 × 10^2^	2.56 × 10^−6^	3
Fab_CH1IgA Y211K	3.69 × 10^5^	8.53 × 10^−4^	2.31 × 10^−9^	8.97 × 10^2^	2.31 × 10^−6^	2

SE: standard errors, U-value: uniqueness value.

### 3.4 Analysis of the Fab_CH1IgA variant

**Fig. 4:**
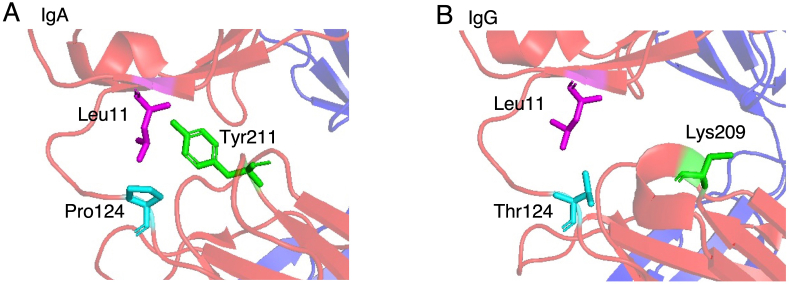
Three-dimensional structures of Fab. The elbow region connecting the V and C domains of the heavy chain is shown for human IgA1 Fab (PDB ID: 3QNY) (A) and for the IgG1-type adalimumab Fab (PDB ID: 4NYL) (B). Residue numbering in IgA1 Fab structure corresponds to that of the Fab variants constructed in this study.

The region near the elbow connecting the V and C domains of the Fab heavy chain in the X-ray crystal structure of IgA, is shown in Fig. 4A. In the IgA Fab, Pro124 positioned in the elbow region, formed van der Waals contacts with Leu11 in the V domain and Tyr211 in the C domain. In contrast, in the IgG-type Fab, the amino acid corresponding to Pro124 was Thr, and the residue corresponding to Tyr211 was Lys (Fig. 4B). Based on these differences, two Fab_CH1IgA variants, Fab_CH1IgA P124T (in which Pro124 of Fab_CH1IgA was substituted with Thr) and Fab_CH1IgA Y211K (in which Tyr211 of Fab_CH1IgA was replaced with Lys), were constructed. Thermal stability analysis by DSC revealed that the *T*_m_ of Fab_CH1IgA P124T was 72.7 °C and that of Fab_CH1IgA Y211K was 66.7 °C, which were both lower than that of Fab_CH1IgA. Analysis of antigen-binding activity using SPR showed that the *K*_D_ values of Fab_CH1IgA P124T and Fab_CH1IgA Y211K were 1.89 × 10^−9^ M and 2.31 × 10^−9^ M, respectively, which were both comparable to that of Fab_CH1IgA. These findings demonstrated that Pro124 and Tyr211 in the C domain, which interact with the V domain in Fab_CH1IgA, have distinct roles compared with those in the IgG type and are critical for thermal stability, although they do not significantly affect antigen-binding activity.

## 4 Discussion

Human antibody heavy chains are divided into five classes: IgG, IgA, IgM, IgE, and IgD, each with distinct and well-characterized biophysical properties [[21], [22], [23]]. Most previous studies have focused on the Fc domain, whereas investigations focusing specifically on Fab fragments are relatively scarce. Within the Fab, the heavy chain forms multiple amino-acid-mediated interactions with the light chain. As all heavy-chain classes associate with a light chain to form a dimer, it is possible to replace the C domain of the heavy chain in a Fab fragment with that of another class. By examining the biophysical properties of Fab fragments in which the heavy-chain C domain is replaced with that of another isotype within the same antibody framework, the physical characteristics unique to each isotype can be elucidated. The therapeutic antibody used in this study, adalimumab, belongs to the IgG1 subclass and is among the most extensively used antibody therapeutics. A Fab variant, in which the C domain of the adalimumab heavy chain was replaced with that of IgA, was constructed and expressed in CHO cells. Expression levels reached approximately 50 mg L^−1^, demonstrating that Fab_CH1IgA could be prepared in sufficient quantities for further characterization.

The structural stability of Fab_CH1IgA was comparable to that of the wild-type Fab. Disulfide bonds substantially contribute to the stability of Fab [24]. In the wild-type Fab, an inter-subunit disulfide bond is formed between the C-terminal cysteine of the Fab-heavy chain and light chain. In contrast, Fab_CH1IgA formed an inter-subunit disulfide bond between Cys135 (numbering according to Fab_CH1IgA sequence) of the Fab-heavy chain and C-terminal cysteine of the light chain. These cysteine residues are located in nearly identical spatial positions [25], and the inter-subunit disulfide bond in IgA1 adopted a configuration similar to that observed in the IgG2, IgG3, and IgG4 subtypes. Furthermore, IgA1 contains an additional intramolecular disulfide bond compared with IgG1. Thus, although Fab_CH1IgA and the wild-type Fab had different disulfide bond configurations, their thermal stabilities were nearly identical. Further analysis of the antigen-binding activity revealed that Fab_CH1IgA exhibited an overall binding affinity comparable to that of the wild-type Fab. Although the association (*k*_on_) and dissociation (*k*_off_) rate constants of Fab_CH1IgA differed somewhat from those of wild-type Fab, its equilibrium dissociation constant (*K*_D_) was not significantly different from that of the wild type. Thus, the overall antigen-binding affinity was not markedly affected, with only slight differences in the kinetic parameters. Despite the relatively low sequence homology between the C domains of IgG1 and IgA1, these findings demonstrate that in the adalimumab Fab, the CH1 domain of IgG can be replaced with that of IgA without markedly affecting thermal stability and antigen-binding activity.

The antigen binding activity of an antibody is predominantly governed by the amino acid sequences of the complementarity-determining regions (CDRs) within its V domains. The V domain exhibits inherent conformational plasticity that enables the structural rearrangements essential for precise antigen recognition [[26], [27], [28]]. The domain is connected to the C domains via the elbow region, which is a structurally flexible hinge that permits substantial variation. This conformational plasticity facilitates antigen recognition by optimizing the relative orientations of the V and C domains during binding. Importantly, C domains are not merely passive structural scaffolds; rather, their dynamic interplay with V domains modulates both antigen-binding affinity and overall structural stability. Sheng et al. revealed that the framework and elbow region somatic mutations reshaped the interdomain geometry between the V and C domains, leading to enhanced antigen affinity and improved structural stability [29]. According to a previous report, within the region near the elbow connecting the V and C domains of the IgA Fab heavy chain, Pro124 (residue numbering based on Fab_CH1IgA) was identified as a constituent of the local hydrophobic core bridging these domains [30]. This hydrophobic core was further stabilized by a hydrogen bond between the hydroxyl group of Tyr211 and main-chain nitrogen atom of Leu11 (residue numbering based on Fab_CH1IgA) [30]. Comparison with the corresponding residues in the IgG-type Fab revealed that Leu11 is conserved, whereas the residue equivalent to Pro124 is Thr and that corresponding to Tyr211 is Lys. In the present study, IgA variants in which these positions are substituted with the IgG-type residues exhibited decreased structural stability. These findings indicate that, in the adalimumab Fab variants examined in this study, interactions between the V and C domains are mediated by the cooperative packing of multiple residues and that the nature of this packing differs between IgA and IgG. Consequently, even a single amino acid substitution may lead to reduced structural stability. Notably, although the sequence identity was not particularly high, Fab_CH1IgA retained structural stability and antigen-binding activity comparable to that of the wild-type Fab. These results suggest that the amino acid residues involved in the V–C domain interactions function as part of a cooperative packing network rather than as independent contributors. In this study, the biophysical properties of the IgA Cα1 domain were investigated for the first time by constructing an IgG–IgA chimeric Fab based on the adalimumab Fab and comparing it with the IgG CH1 domain. Despite the relatively low sequence homology between the parental domains, the chimeric Fab preserved both thermal stability and antigen-binding affinity, highlighting the unique structural contribution of the IgA Cα1 domain. These findings provide important insights for the construction of chimeric antibodies in which the V domain of IgG is fused to the C domain of IgA, as well as for the development of chimeric Fab fragments as novel antibody formats. Moreover, the preservation of both stability and antigen-binding activity despite this sequence divergence highlights the feasibility of converting useful IgG antibodies into IgA-type chimeras. This provides a valuable strategy for antibody engineering, facilitating the development of IgA-based therapeutics. Building on these insights, our results offer valuable guidance for Fab engineering strategies aimed at maintaining thermal stability and antigen-binding activity, while preserving the intrinsic biophysical integrity of the Fab. Altogether, this work can direct further Fab engineering efforts and show potential for practical application in the design of antibody therapeutics with improved structural stability and antigen-binding properties.

## CRediT authorship contribution statement

**Rara Sugimoto:** Writing – original draft, Validation, Methodology, Investigation, Data curation, Conceptualization. **Hitomi Nakamura:** Writing – review & editing, Validation, Methodology, Investigation. **Masato Kiyoshi:** Writing – review & editing, Methodology, Investigation. **Akiko Ishii-Watabe:** Writing – review & editing, Methodology. **Naoko Oda-Ueda:** Writing – review & editing, Project administration. **Takatoshi Ohkuri:** Writing – review & editing, Writing – original draft, Validation, Supervision, Project administration, Methodology, Investigation, Conceptualization.

## Funding

This research did not receive any specific grant from funding agencies in the public, commercial, or not-for-profit sectors.

## Declaration of competing interest

The authors declare that they have no known competing financial interests or personal relationships that could have appeared to influence the work reported in this paper.

## Figures and Tables

**figs1:**
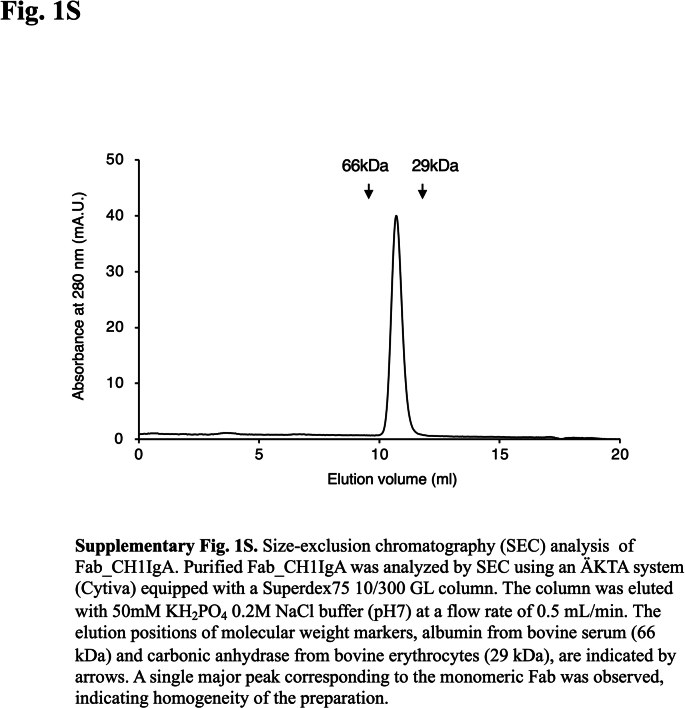


**figs2:**
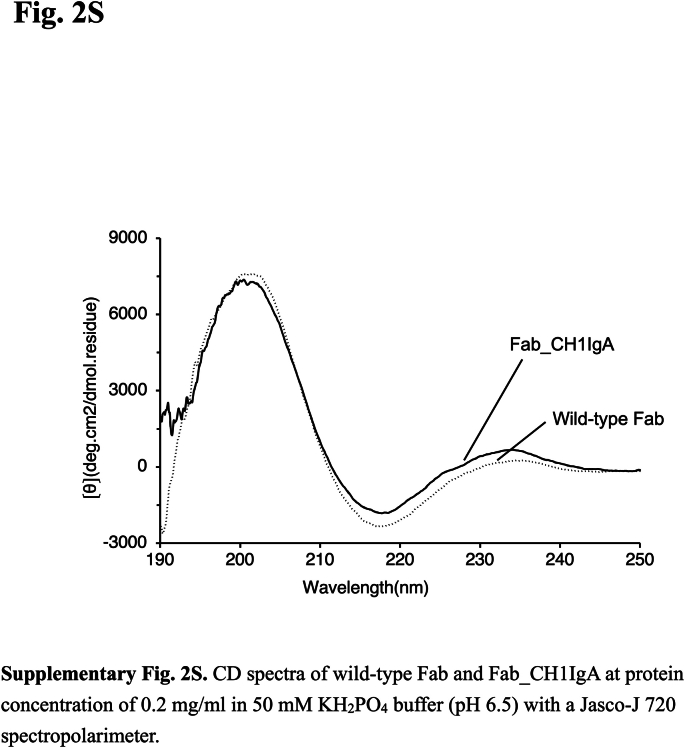


## Data Availability

No data was used for the research described in the article.
